# A Novel Cross-Kingdom Regulatory Mode of Thermostable miR166 from *Taraxacum mongolicum* Inhibits HepG2 Cell Proliferation in Association with Downregulation of the Candidate Target *STAP2*

**DOI:** 10.3390/foods15193470

**Published:** 2026-09-28

**Authors:** Danli Yan, Junrui Wu, Lin Shi, Tong Li, Xinyu Li, Yuxin Wang, Ibtisam Humaidan Alnemari, Hui Li, Jian Zang, Rina Wu

**Affiliations:** 1 College of Food Science, Shenyang Agricultural University, Shenyang 110866, China; 18309861592@163.com (D.Y.); junruiwu@126.com (J.W.); shilin@syau.edu.cn (L.S.); lt171827@163.com (T.L.); xltqmrc@163.com (X.L.); 19999538160@163.com (Y.W.); bes7876@hotmail.com (I.H.A.); lihui920120@163.com (H.L.); 2 Liaoning Engineering Research Center of Food Fermentation Technology, Shenyang Agricultural University, Shenyang 110866, China; 3 Shenyang Key Laboratory of Microbial Fermentation Technology Innovation, Shenyang Agricultural University, Shenyang 110866, China; 4 Northeast China Innovation and Utilization Research Center for Germplasm Resources (Analysis and Testing Center), Shenyang Agricultural University, Shenyang 110866, China

**Keywords:** anti-cancer, cross-kingdom regulation, miRNA, *STAP2*, *Taraxacum mongolicum*

## Abstract

*Taraxacum mongolicum* contains various bioactive components. This study investigated the potential of thermostable microRNAs (miRNAs) derived from this plant as cross-kingdom regulators and evaluated their anti-proliferative effects on HepG2 cells. High-throughput sequencing was used to screen thermostable miRNAs and predict their target genes. Enrichment and target gene expression in gavaged mice were analyzed to assess cross-kingdom regulatory potential. Cellular assays evaluated anti-proliferative activity. miR166 was identified as a thermostable miRNA in *T. mongolicum*, and bioinformatics indicated its possible involvement in cancer-associated mucin-type O-glycan biosynthesis. RT-qPCR revealed that miR166 was negatively correlated with signal transducing adaptor family member 2 (*STAP2*) mRNA expression. In mice, administration of total RNA extracted from fresh *T. mongolicum* or its water decoction led to significant changes in miR166 levels in stomach, blood, liver, spleen, and intestine, accompanied by reduced *STAP2* mRNA, suggesting cross-kingdom regulation. Overexpression of miR166 induced a delay in G1/S transition in HepG2 cells in a concentration-dependent manner, accompanied by reduced *STAP2* expression and decreased cell viability. These findings provide preliminary experimental evidence that thermostable miR166 from *T. mongolicum* is associated with inhibition of HepG2 proliferation. They also suggest *STAP2* as a candidate regulatory target, offer a potential new molecule for further investigation in liver cancer intervention, and provide a preliminary molecular basis for the medicinal and edible value of *T. mongolicum*.

## 1. Introduction

*Taraxacum mongolicum* is a common plant that is edible, nutritious and widely used for medicinal purposes. It has been demonstrated to possess antibiotic properties and has consequently become a popular natural health food [1]. A number of experimental studies have demonstrated that *T. mongolicum* exhibits a range of pharmacological effects, including antibacterial, antiviral, antitumour, anti-inflammatory and pain-relieving properties, as well as the treatment of metabolic diseases. Additionally, the nutrients present in *T. mongolicum*, which can be considered a functional food, are widely regarded as key factors in regulating the expression of nutritional genes in animals. These remarkable effects are primarily attributed to the high concentration of active compounds in *T. mongolicum*, including flavonoids, polysaccharides, sterols, terpenoids, and pigments [2,3]. These compounds have been demonstrated to possess significant inhibitory effects on a range of cancers [4,5,6]. It is worth noting that *T. mongolicum* extract shows considerable potential as an adjunct treatment for liver diseases, and its bioactive components have therefore attracted ongoing attention. However, most existing studies have focused on the plant’s small-molecule chemical constituents; there are currently no reports on whether *T. mongolicum* contains non-small-molecule endogenous active components, such as miRNAs, or whether such molecules are involved in its pharmacological effects.

miRNAs are short endogenous single-stranded non-coding RNAs of 18–24 nucleotides that regulate gene expression. miRNAs are involved in virtually all key biological processes, including embryonic development, cell differentiation, apoptosis, metabolism, and immune responses. Abnormal miRNA expression is closely associated with the onset and progression of various diseases, such as Alzheimer’s disease, Parkinson’s disease, cardiovascular disease, diabetes, and cancer [7,8,9]. Beyond endogenous roles, a growing body of evidence indicates that plant-derived miRNAs can survive the gastrointestinal tract, enter the circulation, and modulate mammalian gene expression in a cross-kingdom manner. Following consumption of rice, miR168a derived from rice is stably absorbed, significantly enriched in serum and liver, and has been shown to downregulate hepatic low-density lipoprotein receptor adapter protein 1 (*LDLRAP1*) mRNA, thereby disrupting lipoprotein metabolism and elevating hepatic low-density lipoprotein levels [10,11,12]. This finding demonstrates that plant miRNAs, via dietary intake, can exert gene regulatory effects in mammalian liver. Beyond rice, multiple plant miRNAs have been shown to possess cross-kingdom bioactivity. Honeysuckle-derived miR2911, which exhibits high thermostability, inhibits the replication of H1N1 and H5N1 influenza viruses by targeting PB2 and NS1 proteins, thereby reducing the lethality of virus-infected mice [13]. Lettuce-derived artificial miRNAs (amiRNAs) targeting hepatitis B surface antigen (HBsAg) gene have been engineered using the plant’s endogenous miRNA biogenesis machinery; when fed to transgenic mice, these amiRNAs crossed the gastrointestinal barrier, entered the bloodstream and liver, and suppressed HBsAg expression, alleviating liver injury without observable adverse effects [14]. These findings have transformed our understanding of functional foods, suggesting that plant miRNAs may represent a new class of bioactive components with therapeutic potential.

*T. mongolicum* is commonly administered as a hot-water decoction in traditional practice. Yet whether thermostable miRNAs in this plant can survive such processing and exert cross-kingdom regulatory effects on human genes remains completely unknown. In this study, we hypothesized that thermostable miRNAs in *T. mongolicum* might persist after decoction and act as cross-kingdom regulators of cancer-relevant targets. To test this, we employed high-throughput sequencing to screen for thermostable miRNAs, bioinformatics to predict their human target genes, and both in vivo and in vitro models to evaluate their uptake, target modulation, as well as to assess their anti-proliferative activity. The results identify miR166 as a thermostable miRNA that correlates with reduced *STAP2* mRNA expression and suppressed HepG2 cell viability, providing initial experimental evidence for a novel cross-kingdom regulatory mode of *T. mongolicum*-derived miRNA and offering a molecular basis for the medicinal value of this plant beyond its conventional phytochemicals.

## 2. Materials and Methods

### 2.1. Preparation of T. mongolicum and Its Decoction

Fresh *T. mongolicum* (FT) (flower-free above-ground parts) was purchased from a local market in Shenyang, China, and taxonomically authenticated. After soaking 30 g of FT in 300 mL of distilled water for 30 min, the soaked material was brought to a boil and maintained at a boil for 30 min. The resulting mixture was filtered to obtain the primary filtrate, and the residue was subjected to a second extraction with the same volume of distilled water under identical conditions. The two filtrates were combined and concentrated to 15 mL using a rotary evaporator to a final concentration of 2 g/mL, referred to as the decoction of *T. mongolicum* (DT). The DT was stored at −80 °C until further use.

### 2.2. High-Throughput Sequencing and Bioinformatics Approaches

Small RNAs for high-throughput sequencing were isolated from 50 mg of FT or 250 μL of DT using the Universal Plant MicroRNA Kit (Bioteke, Beijing, China) according to the manufacturer’s instructions. High-throughput sequencing and bioinformatics of RNA samples were performed by Personalbio (Shanghai, China). After removing the adaptor sequences from the raw data, the clean reads were aligned against the Rfam and miRBase databases to obtain the annotation information of various small RNAs. The expression of miRNAs was statistically analyzed using counts per million (CPM) values. High-throughput sequencing and bioinformatics for FT and DT were independently repeated and verified by qRT-PCR. The raw sequencing data have been deposited in the NCBI SRA database under BioProject accession number PRJNA1533588.

### 2.3. Cell Culture

HEK293T and HepG2 cells were maintained in a class 10,000 cell culture room at the College of Food Science, Shenyang Agricultural University. HEK293T cells were obtained from the Institute of Chinese Academy of Sciences, Beijing, China. HepG2 cells were provided by the laboratory of the College of Animal Husbandry and Veterinary Medicine, Shenyang Agricultural University. Cells were cultured in Dulbecco’s Modified Eagle Medium (DMEM, Sale EI Chemical Reagent Co., Ltd., Shenyang, China) supplemented with 10% fetal bovine serum (FBS, Sale EI Chemical Reagent Co., Ltd., Shenyang, China) and 1% penicillin-streptomycin solution (100 U/mL penicillin and 100 μg/mL streptomycin, Sale EI Chemical Reagent Co., Ltd., Shenyang, China, at 37 °C in a 5% (*v*/*v*) CO_2_ atmosphere.

### 2.4. Animal Feeding

Male ICR mice (*n* = 6 per group) were purchased from SiPeiFu (Beijing, China), housed under specific pathogen-free conditions with a 12 h light/dark cycle and free access to water and standard chow, and fasted overnight prior to gavage. Mice were randomly divided into four groups (*n* = 6 per group): the decoction group (0.15 mg/mL decoction), the RNase group (DT treated with RNase A (Solarbio, Beijing, China) at 100 µg/mL, 37 °C for 30 min followed by heat inactivation at 65 °C for 10 min prior to gavage, complete removal of RNA was confirmed by agarose gel electrophoresis, Figure S1), the Total RNAs (1.5 mg/mL FT Total RNA), and the Control group (phosphate-buffered saline, PBS) (Solarbio, Beijing, China). Each gavage was 0.2 mL at two-hour intervals, and a total of five gavages were administered. At 1 h, 3 h, 6 h, and 12 h after the final gavage, mice were euthanized; tissues (stomach, intestine, liver, and spleen) and blood were collected, and all samples were stored at −80 °C until further analysis. All animal operating procedures were approved by the Experimental Animal Welfare Ethics Review Committee of Shenyang Agricultural University on 2 December 2020 (IACUC Issue No.: 2020120101).

### 2.5. RNA Extraction and qRT-PCR

Small RNAs (<100 nt) from FT and DT were extracted using the Universal Plant MicroRNA Kit (Bioteke, Beijing, China). Total RNAs extracted from mouse blood and other tissues and cells were extracted with Trizol reagent (Leagene, Beijing, China). Total RNAs were reverse transcribed into cDNAs using the miRcute Plus miRNA First-Strand cDNA Kit (Tiangen, Beijing, China). qRT-PCR was performed using miRcute Plus miRNA qPCR Kit (SYBR Green) (Tiangen, Beijing, China) according to the manufacturer’s instructions. miR166 levels were normalized to U6 snRNA, and relative expression was calculated using the 2^−ΔΔCt^ method. All assays were performed with three independent biological replicates, and each sample was analyzed in three technical replicates. The primers based on the original sequence of miR166 and the internal reference U6 were designed and synthesized by Tiangen Biochemical Technology Co. (Beijing, China). Primers for the quantitative real-time PCR and reverse transcription of signal transducing adaptor family member 2 (*STAP2*), OTU deubiquitinase 7A (*OTUD7A*), DLG associated protein 2 (*DLGAP2*), polypeptide N-acetylgalactosaminyltransferase 14 (*GALNT14*), bone morphogenetic protein 10 (*BMP10*), and glyceraldehyde-3-phosphate dehydrogenase (*GAPDH*) were synthesized by Bgi Genomics Co., Ltd. (Beijing, China), and the sequences of primers used are listed as Table 1. All PCRs were performed in triplicate. The relative amount of transcripts was calculated using the 2^−ΔΔCt^ formula. The data analysis was performed using ABI Prism 7500 SDS Software (Applied Biosystems, Thermo Fisher Scientific, Waltham, MA, USA).

### 2.6. Cell Transfection

The miR166 mimics (5′-UCGGACCAGGCUUCAUUCCUU-3′) and negative control were purchased from Ribobio (Guangzhou, China). Oligonucleotide transfection was performed with riboFECT™ CP Reagents (Ribobio, Guangzhou, China). HEK293T and HepG2 were transfected using riboFECT™ CP Reagent (Ribobio, Guangzhou, China) according to the instructions. miR166 was transfected at 25 nM, 50 nM and 100 nM. After 72 h of transfection, cells were harvested and subjected to qRT-PCR, cell activity and cycle analysis. Transfection efficiency was validated by qRT-PCR detection of mature miR166 levels in transfected cells compared to the negative control.

### 2.7. Cell Viability Assay

HepG2 cells (1 × 10^4^ cells/well) were seeded in 96-well plates and transfected with miR166 mimics at 25, 50, or 100 nM, or negative control mimics. After 72 h, cell viability was assessed by Wu’s method [15]. Cells were incubated with 3-(4,5-dimethylthiazol-2-yl)-2,5-diphenyltetrazolium bromide (MTT; Solarbio, Beijing, China) (5 mg/mL) for 4 h, formazan crystals were dissolved in dimethyl sulfoxide (DMSO; Solarbio, Beijing, China), and absorbance was measured at 490 nm.

### 2.8. Cell Cycle Analysis

HepG2 cells (4 × 10^6^ cells/well) were seeded into a 6-well plate with different concentrations of miR166 (25, 50 and 100 nM) to study cell cycle progression. 72 h later, cells were harvested, fixed, stained with propidium iodide, and subjected to flow cytometry analysis for cell-cycle distribution.

### 2.9. Statistical Analysis

All data from the experiments were tested in triplicate and expressed as the mean ± standard deviation. All analyses were performed using the SPSS Statistics 22.0 and GraphPad Prism 9.0. Two groups were analyzed by Student’s *t*-test. Differences among groups were analyzed by one-way ANOVA. Significance was accepted at *p* ≤ 0.05.

## 3. Results

### 3.1. Identification of Thermostable miRNAs in T. mongolicum by High-Throughput Sequencing

The results of high-throughput sequencing of small RNAs showed that the lengths of fresh *T. mongolicum* (FT) samples were predominantly distributed at 21 nt and 24 nt in clean total reads and clean unique reads, respectively, and the sequence lengths in *T. mongolicum* decoction (DT) samples were concentrated at 18 nt and 24 nt, respectively (Figure 1). Since miRNAs are typically 18–25 nt in length, these results indicated that the majority of the discovered small RNAs in *T. mongolicum* are miRNAs. The sequencing data also exhibited typical miRNA base preferences (Supplementary Figure S2).

After annotation against the miRBase database, 419 miRNA species were identified in FT samples, whereas only 56 miRNAs were detected in DT samples. Among the miRNAs detected in both FT and DT, miR166, miR159, miR158, miR398, and miR156 were the most abundant in the decoction, suggesting their potential thermostability. Notably, miR166 was highly expressed in both FT and DT, whereas many other miRNAs were substantially reduced or completely degraded after boiling (Figure 2), likely due to high-temperature exposure and the complex chemical environment during decoction, which may have caused structural alterations or direct degradation of miRNAs.

The relative expression of miR166 in FT and DT was further validated by qRT-PCR. The results showed that the miR166 level in FT was approximately 29.6-fold higher than that in DT (Figure 3), which was consistent with the sequencing data and further supported the presence of thermostable miR166 in *T. mongolicum*. This provided a basis for subsequent functional studies of miR166.

### 3.2. GO and KEGG Enrichment Analysis of miR166

To explore the potential functions of miR166, the human genome was used as the reference, and miRanda software was employed to predict potential target genes of thermostable miR166 in *Homo sapiens*. The predicted target genes were then subjected to Gene Ontology (GO) and Kyoto Encyclopedia of Genes and Genomes (KEGG) pathway enrichment analysis. GO analysis showed that the predicted target genes were mainly localized in the integral component of the synaptic membrane and enriched in molecular functions such as phosphatidylinositol 3-kinase (PI3K) binding, ubiquitin-conjugating enzyme (E2C) binding, and transmembrane receptor protein tyrosine kinase activity, suggesting their possible involvement in calcium ion transmembrane transport, activation of adenylate cyclase activity, and regulation of synaptic vesicle transport (Figure 4A). KEGG pathway enrichment analysis revealed that the predicted target genes were most significantly enriched in mucin-type O-glycan biosynthesis, followed by regulation of lipolysis in adipocytes and the cGMP-PKG signaling pathway (Figure 4B).

### 3.3. Screening and Validation of miR166 Target Genes

To identify potential cancer-related targets of thermostable miR166, miRanda software was used to predict target genes in the human genome. Among the top-ranked candidates with a score ≥150, five genes, *STAP2*, *OTUD7A*, *DLGAP2*, *GALNT14*, and *BMP10*, were selected for further validation by qRT-PCR. HEK293T cells were transfected with miR166 mimics, and the relative expression levels of these five candidate genes were measured. Among them, only *STAP2* expression was significantly reduced after miR166 transfection (Figure 5). Notably, although *GALNT14* was among the predicted targets, its expression was not significantly altered in our qRT-PCR validation. These results suggested that not all bioinformatically predicted targets were regulated by miR166 at the mRNA level under our experimental conditions. Therefore, *STAP2* was selected as a candidate target for subsequent functional studies, primarily because it was the only gene showing consistent downregulation in response to miR166 and has been implicated in tumor-related signaling. Although it was not among the top KEGG-enriched pathways, the experimental evidence and supporting literature made it the most promising candidate for further investigation.

### 3.4. Cross-Kingdom Transport of miR166 from T. mongolicum and Its Regulatory Effects on Gene Expression in Mice

Mice were gavaged with PBS, *T. mongolicum* decoction, RNase-treated decoction, or FT total RNA. Total RNA was extracted from the stomach, intestine, blood, heart, liver, spleen, kidney, and brain of mice belonging to four distinct groups at 1 h post-administration. No significant difference in miR166 levels was observed between the RNase-treated decoction group and the PBS control group (Figure 6A–H). Complete removal of RNA in the RNase-treated decoction was confirmed by agarose gel electrophoresis (Figure S1). These results suggested that the changes in relative miR166 content were attributable to miR166 derived from *T. mongolicum*. The relative miR166 content in the brain, heart, and kidney showed no significant changes compared to the control (Figure 6C,D,G). However, relative miR166 levels were elevated in the stomach, blood, liver, spleen, and small intestine of mice in both the decoction and FT total RNA groups compared to the PBS control group (Figure 6A,B,E,F,H), indicating that miR166 was absorbed into these tissues. Notably, miR166 levels in these organs were slightly higher in the decoction group than in the FT total RNA group. Simultaneously, we observed that the *Stap2* gene was expressed in several organs of mice. Determining the relative levels of *Stap2* in blood, liver, spleen, and intestine of mice after drug administration by qRT-PCR, we found that *Stap2* expression was down-regulated in certain tissues of mice with high miR166 levels (Figure 7A–D).

Given the more pronounced enrichment of miR166 in the stomach, blood, liver, spleen, and intestines 1 h post-gavage of the *T. mongolicum* decoction, mice from the *T. mongolicum* decoction group were subsequently selected to investigate the metabolic process of miR166. The kinetics of miR166 in various tissues were observed at 1, 3, 6, and 12 h post-gavage, and Figure 8 showed the curves of the relative expression levels of miR166 in different tissues. Similar trends were observed in the metabolic process of miR166 following gavage with total RNA extracted from fresh *T. mongolicum*. In the stomach, the relative expression level of miR166 consistently decreased from 1 to 12 h, showing a decrease of (1.31 ± 0.18) ×10^−6^ -fold at 12 h compared to 1 h (Figure 8A). The relative expression levels of miR166 in plasma, liver and spleen exhibited a similar trend, peaking at 3 h and subsequently declining. Compared to 1 h post-gavage, the relative expression level of miR166 in plasma was increased by 3.98 ± 0.39-fold at 3 h and decreased to 0.95 ± 0.11-fold at 12 h (Figure 8B). In the liver, miR166 enrichment was 13.27 ± 2.59-fold higher at 3 h than at 1 h, decreasing to 6.18 ± 0.31-fold at 6 h and 2.99 ± 0.47-fold at 12 h (Figure 8C). The relative expression level of miR166 in the spleen was 3441.33 ± 267.54-fold higher at 3 h post-gavage than at 1 h (Figure 8D). In the intestine, miR166 enrichment showed an initial increase followed by a decline, peaking at 6 h, with a relative expression level markedly elevated compared to 1 h (Figure 8E). However, because miR166 levels in the intestine of the control group were extremely low and close to the detection limit, this fold-change should be interpreted with caution. The absolute fold-change may be influenced by the low baseline in the control group.

### 3.5. Effects of miR166 on HepG2 Cell Viability and Cell Cycle

The relative miR166 expression levels in the 25 nM, 50 nM, and 100 nM groups were 33.53-fold, 58.13-fold, and 81.54-fold higher than the NC group, respectively, confirming successful and concentration-dependent delivery of miR166 mimics (Figure S3). To evaluate the biological effects of miR166 on HepG2 cells, cells were transiently transfected with miR166 mimics at 25 nM, 50 nM, and 100 nM, with negative control (NC) mimics as the reference. After 72 h, cell viability was assessed by MTT assay (Figure 9A). Compared with the NC group, the relative cell viability in the 25 nM, 50 nM, and 100 nM miR166 groups was 92.61% ± 5.06%, 71.11% ± 2.86%, and 64.49% ± 2.08%, respectively. The above results indicated that the survival rate of HepG2 cells significantly decreased with increasing miR166 transfection concentrations.

Cell cycle analysis revealed that the proportion of G1-phase cells significantly decreased, while the proportion of S-phase cells increased, after miR166 treatment compared with the control group (*p* < 0.05) (Figure 9B), suggesting a delay in G1/S transition rather than G2 phase arrest. Furthermore, *STAP2* expression was significantly reduced with increasing miR166 concentrations (Figure 9C), suggesting a potential association between miR166-mediated *STAP2* downregulation and the observed reduction in cell viability.

## 4. Discussion

Dietary-derived plant miRNAs have emerged as a novel class of bioactive molecules capable of cross-kingdom regulation in mammals. For example, miR159 derived from broccoli can be transported to various tissues and cells via extracellular vesicles and eventually accumulate in breast tissue. Experimental results have verified that miR159 inhibits tumor growth by targeting and binding to *TCF7*, thereby reducing the protein expression level of MYC [16]. In addition to plant miRNAs, dietary miRNAs from other sources can also be functionally active in recipients. Human milk-derived miRNAs, including miR-148a-3p, miR-30b-5p, miR-182-5p and miR-200a-3p, all of which exhibited immunomodulatory functions and promoted the aggregation of *Bifidobacterium* in newborns [17]. This supports the broader concept that dietary miRNAs can survive and function in mammals, which is consistent with the cross-kingdom regulation observed in our study. miRNAs from *Salvia miltiorrhiza* (Sal-miR-1 and Sal-miR-3) regulate the OTUD7B/KLF4/NMHC IIA axis in mammalian cells and inhibit carotid ligation-induced intimal hyperplasia and suppress thrombin-induced VSMC migration and monocyte adhesion to VSMCs [18]. *Atropa belladonna*-derived miR-9497 targets the 3′-UTR of *ZNF-691* mRNA and downregulates its expression. This interaction may contribute to the neurotoxic and psychoactive properties of *A. belladonna* by modulating *ZNF-691*-sensitive genes [19]. Similarly, levels of *Gastrodia elata*-derived miRNAs (Gas-miR01 and Gas-miR02) were detected in the serum, liver, brain, kidney and spleen of mice following decoction administration, and were associated with reduced expression of the NF-κB-driven feedback regulator A20 [20]. These findings support the concept that plant miRNAs can be absorbed through dietary intake and exert regulatory functions in recipients, highlighting their potential as therapeutic agents.

*T. mongolicum* has a long history of medicinal use and is commonly consumed as a decoction in China [21]. In this study, we investigated whether thermostable miRNAs in *T. mongolicum* could survive decoction and exert cross-kingdom regulatory effects. High-throughput sequencing identified miR166 as one of the most abundant thermostable miRNAs in the decoction, leading to its selection for further investigation. Bioinformatics analysis predicted that miR166 targets genes enriched in pathways associated with cancer, including the PI3K/Akt/mTOR signaling pathway, which is known to regulate cell growth, proliferation, and apoptosis in various malignancies [22]. In addition, miR166 target genes were predicted to be enriched in the ubiquitin-coupled enzyme pathway, which is involved in cell division and protein degradation [23,24]. Bioinformatic predictions showed that *T. mongolicum*-derived miR166 regulates mucin-type O-glycans biosynthesis. Aberrant O-glycosylation is strongly correlated with tumor progression and serves as a clinical tumor biomarker; structurally specific core 1 and core 2 O-GalNAc glycans can bind tumor-associated dysadherin, suppress its expression, and further elevate E-cadherin levels, thereby modulating tumor metastasis [25,26].

Based on miRanda predictive scores and pathway enrichment analysis, five candidate target genes, including *STAP2*, *OTUD7A*, *DLGAP2*, *GALNT14* and *BMP10* were further screened and experimentally validated to clarify which bioinformatically predicted targets can be effectively regulated by miR166 at the mRNA level. Among these, only *STAP2* showed consistent downregulation upon miR166 overexpression in HEK293T cells, leading to its selection for further functional studies. *STAP2* is expressed in various cell types, including lymphocytes, macrophages, dendritic cells, and hepatocytes, and has been implicated in signal transduction and cytokine regulation through its unique structural domains [27].

In this experiment, the relative levels of *T. mongolicum* miR166 in the intestine, serum, liver, and spleen of mice at 1 h post-administration were significantly increased compared with the control group, suggesting that miR166 from *T. mongolicum* could be absorbed by mice across kingdoms. This finding is consistent with previous reports showing that Gas-miR01 and Gas-miR02 could be absorbed by mice following oral administration of total RNA from *Gastrodia elata* [20]. There was no significant difference between the RNase-treated decoction group and the PBS control group, suggesting that the observed change in the relative content of miR166 in mice was attributable to *T. mongolicum*-derived miRNAs. Time-course analysis revealed that miR166 levels in the serum and other organs exhibited significant changes over time, likely due to the transport of miR166 from the stomach to other tissues. The relative miR166 levels in the intestine peaked at 6 h post-administration, while those in the blood, liver, and spleen all reached maximum levels at 3 h, showing a general tendency to increase and then decline. These results suggest that the transport of miR166 from the stomach to other organs likely occurs via the bloodstream, with peak levels observed at 3 h post-gavage in most tissues, except for the intestine, where peak levels were observed at 6 h.

Based on the observed enrichment of miR166 in the liver and its association with *STAP2* downregulation in vivo, HepG2 cells were selected for functional studies. miR166 overexpression induced a delay in G1/S transition and reduced cell viability in a concentration-dependent manner, accompanied by reduced *STAP2* expression. These results suggest a potential association between miR166-mediated *STAP2* downregulation and the observed anti-proliferative effects. *STAP2* contains an N-terminal PH domain and a C-terminal proline-rich region, which enable its participation in various signaling pathways through interactions with multiple binding partners [28]. Through its PH domain, *STAP2* has been reported to associate with DUSP12 and modulate p38-mediated inflammatory signaling in macrophages. Additionally, the YXXQ motif in the C-terminus of *STAP2* serves as a docking site that binds to the SH2 domain of signal transducer and activator of transcription 3 (STAT3) [29,30]. Elevated STAT3 phosphorylation has been observed in hepatocellular carcinoma specimens and is associated with tumor progression [31]. Given the persistent activation of STAT3 in liver cancer, it is plausible that miR166-mediated downregulation of *STAP2* may influence STAT3-related signaling, although direct evidence for this mechanism requires further experimental validation.

In addition, this study has several limitations. All functional experiments were conducted using a single cell line (HepG2), which restricts the generalizability of the results. The in vivo functional effects were not validated in tumor-bearing mice. Moreover, qRT-PCR-based detection of miR166 in mouse tissues cannot fully distinguish full-length functional miRNAs from degraded fragments or endogenous small RNAs with sequence similarity; future studies using Northern blot or small RNA sequencing are needed to confirm the integrity and identity of the absorbed miR166. The exceptionally high fold-change observed in the intestine may be influenced by the extremely low miR166 levels in the control group, which were close to the detection limit, and detection in tissues supports absorption but does not prove functional regulation in target organs. Direct evidence for *STAP2* targeting by miR166 requires dual-luciferase reporter assays, which were not performed in this study. Finally, the in vitro experiments using synthetic miR166 mimics at relatively high concentrations should be interpreted as proof-of-concept evidence rather than a direct simulation of dietary intake. Therefore, the observed downregulation of *STAP2* and enrichment of miR166 only support a functional association, rather than anti-tumor efficacy. Future studies should use tumor-bearing mouse models and a wider range of cancer cell lines to further validate the anti-proliferative potential of miR166 and its therapeutic significance.

## 5. Conclusions

In this study, we identified miR166 as a thermostable miRNA in *T. mongolicum* and observed its cross-kingdom uptake in mice, where it was associated with downregulation of *STAP2* expression in multiple tissues. In HepG2 cells, miR166 overexpression induced a delay in G1/S transition and reduced cell viability in a concentration-dependent manner, accompanied by reduced *STAP2* expression. These findings suggest that miR166 may represent a candidate bioactive molecule of *T. mongolicum* with anti-proliferative potential. This study provides molecular clues linking thermostable plant miRNAs to cross-kingdom regulation and identifies *STAP2* as a candidate target for further mechanistic studies in liver cancer. Future research could explore the potential of miR166 as a functional food component or therapeutic agent, pending further validation of its efficacy and safety.

## Figures and Tables

**Figure 1:**
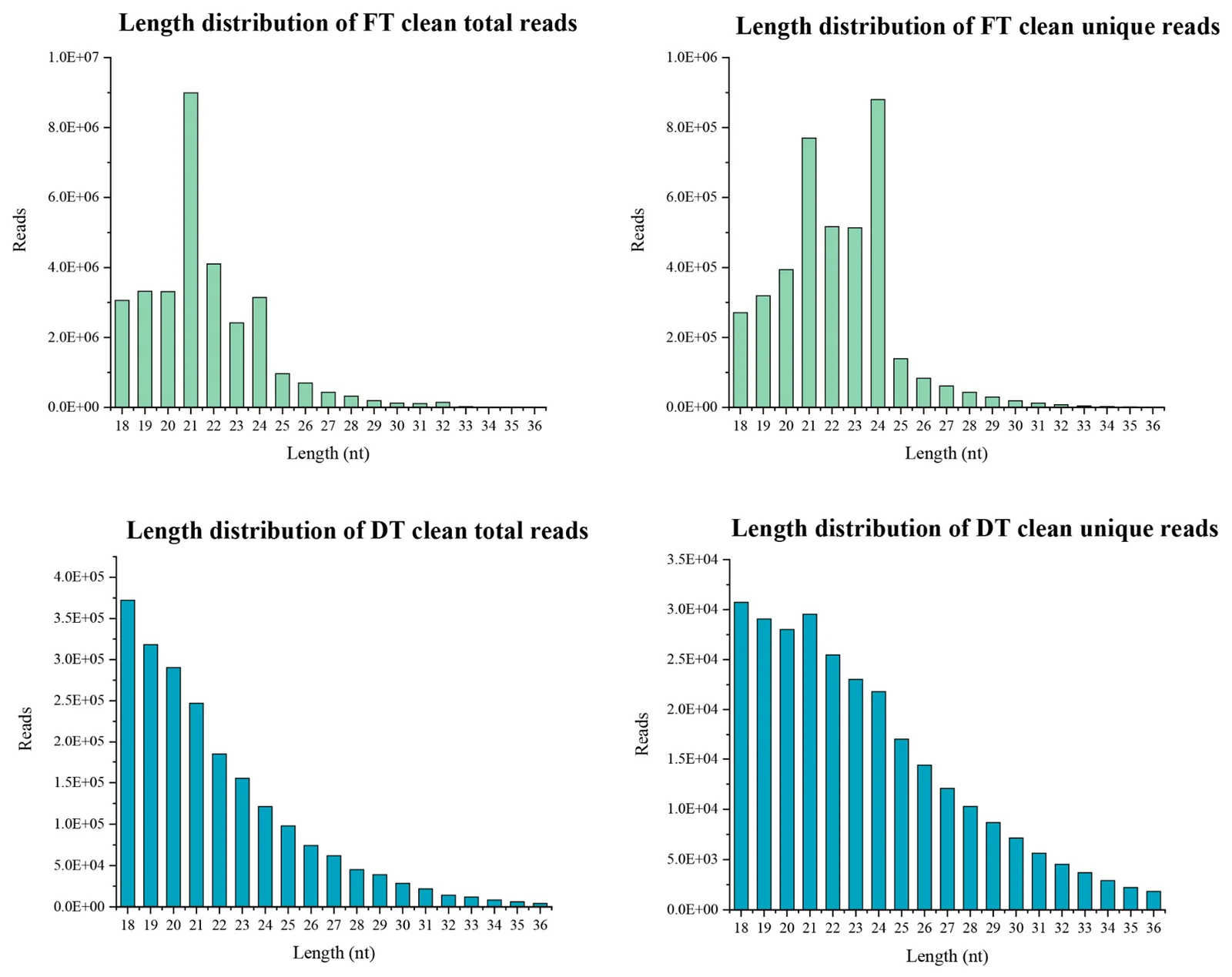
Size distribution of clean sequencing reads from FT (fresh) and DT (decoction) preparations of *T. mongolicum*.

**Figure 2:**
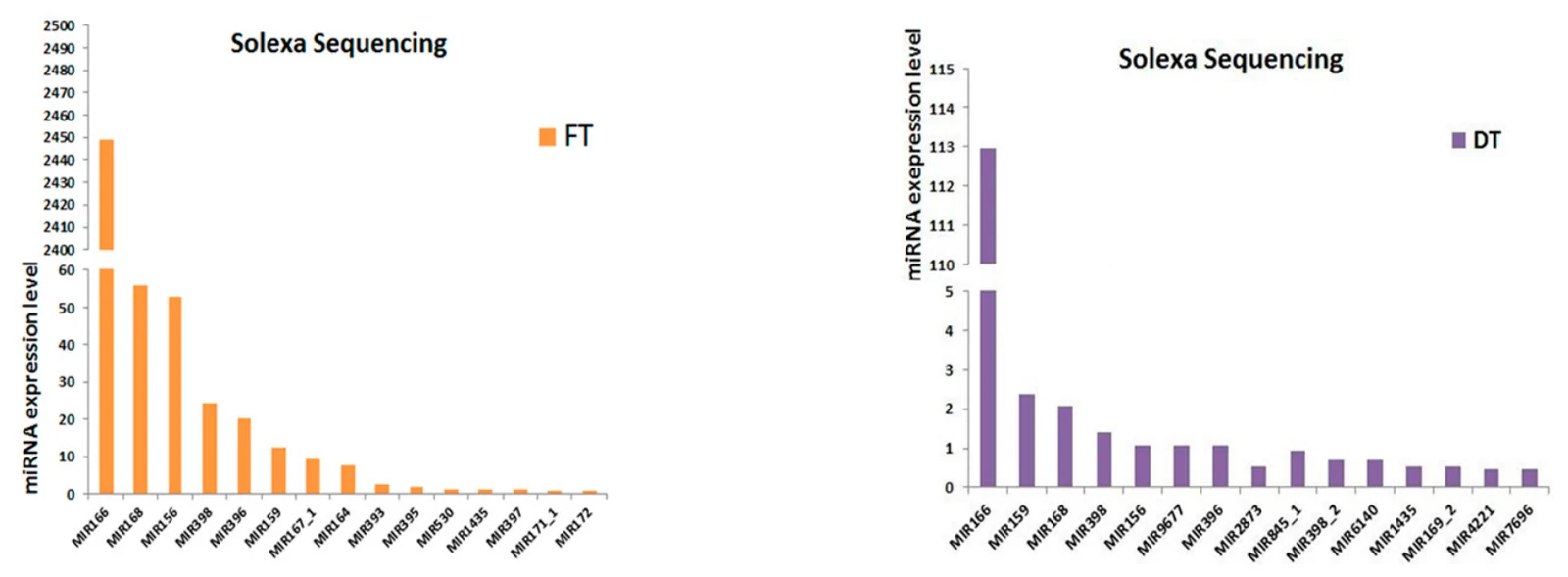
Abundance ranking of the top ten miRNAs in FT and DT samples of *T. mongolicum*, quantified as counts per million (CPM).

**Figure 3:**
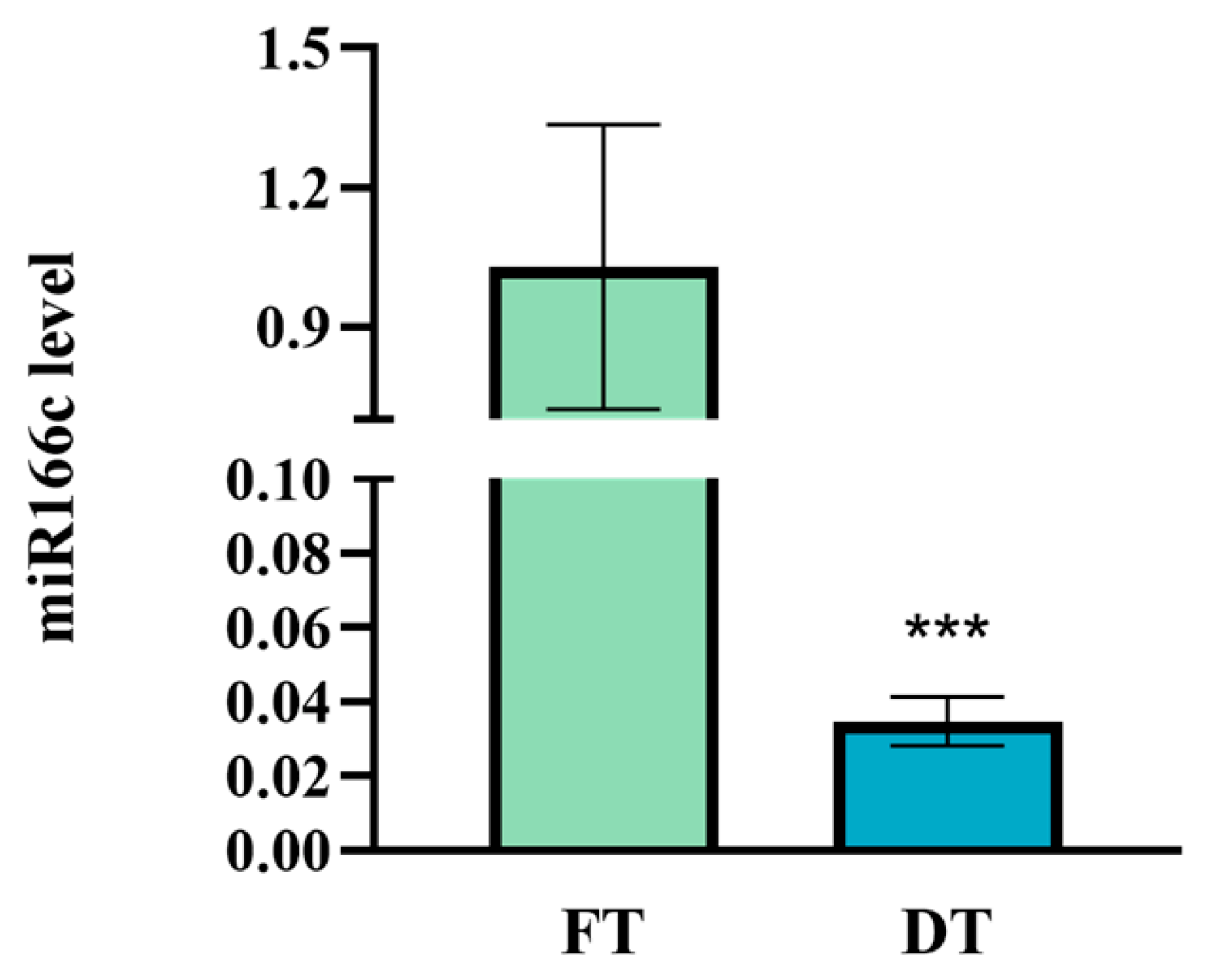
Expression comparison of miR166 between FT and DT preparations of *T. mongolicum* by qRT-PCR. Data are presented as mean ± SD (*n* = 3 biological replicates). Note: *** *p* < 0.001 vs. FT.

**Figure 4:**
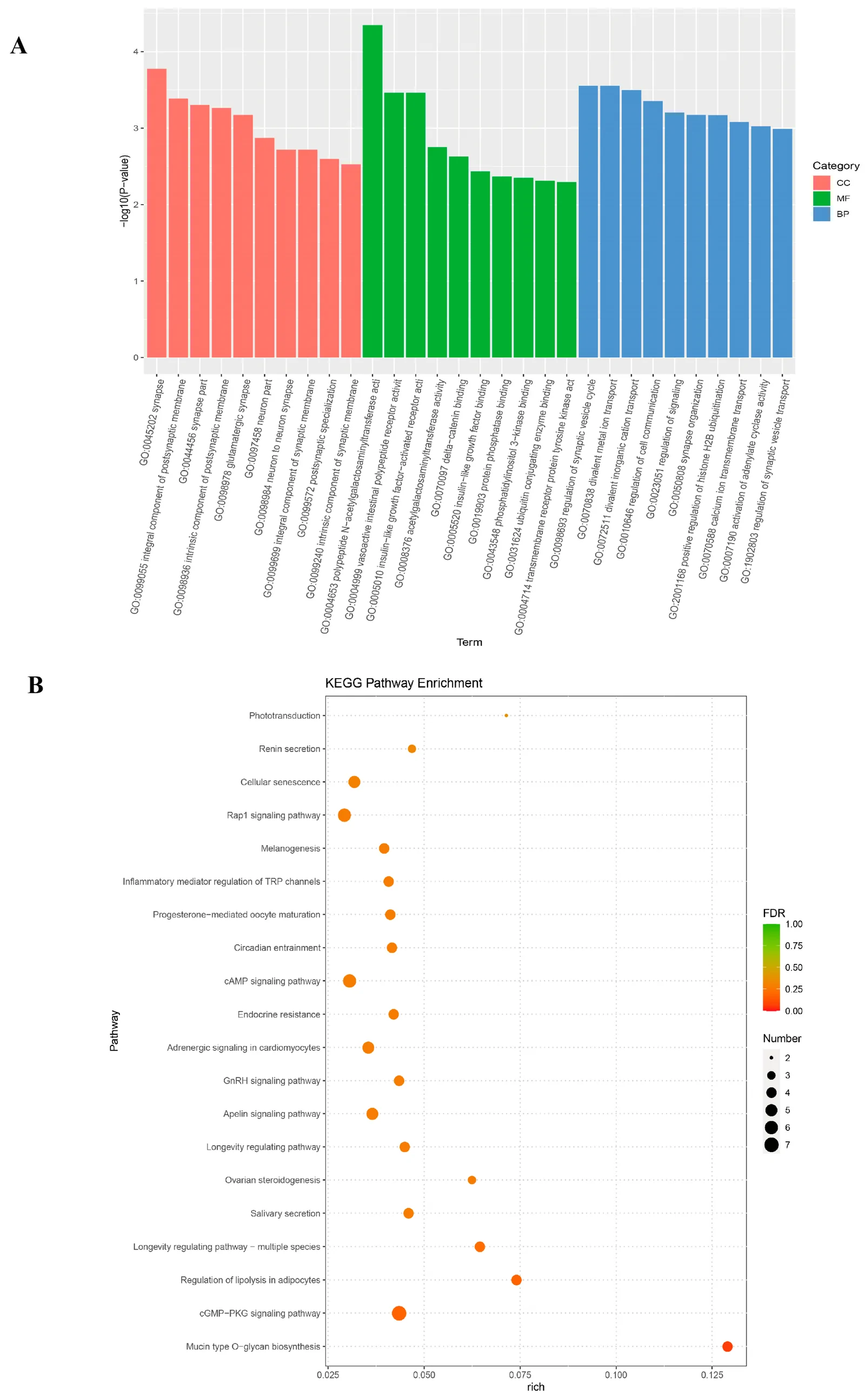
Enrichment analysis of miR166 target genes. (**A**) GO functional classification. (**B**) KEGG pathway enrichment.

**Figure 5:**
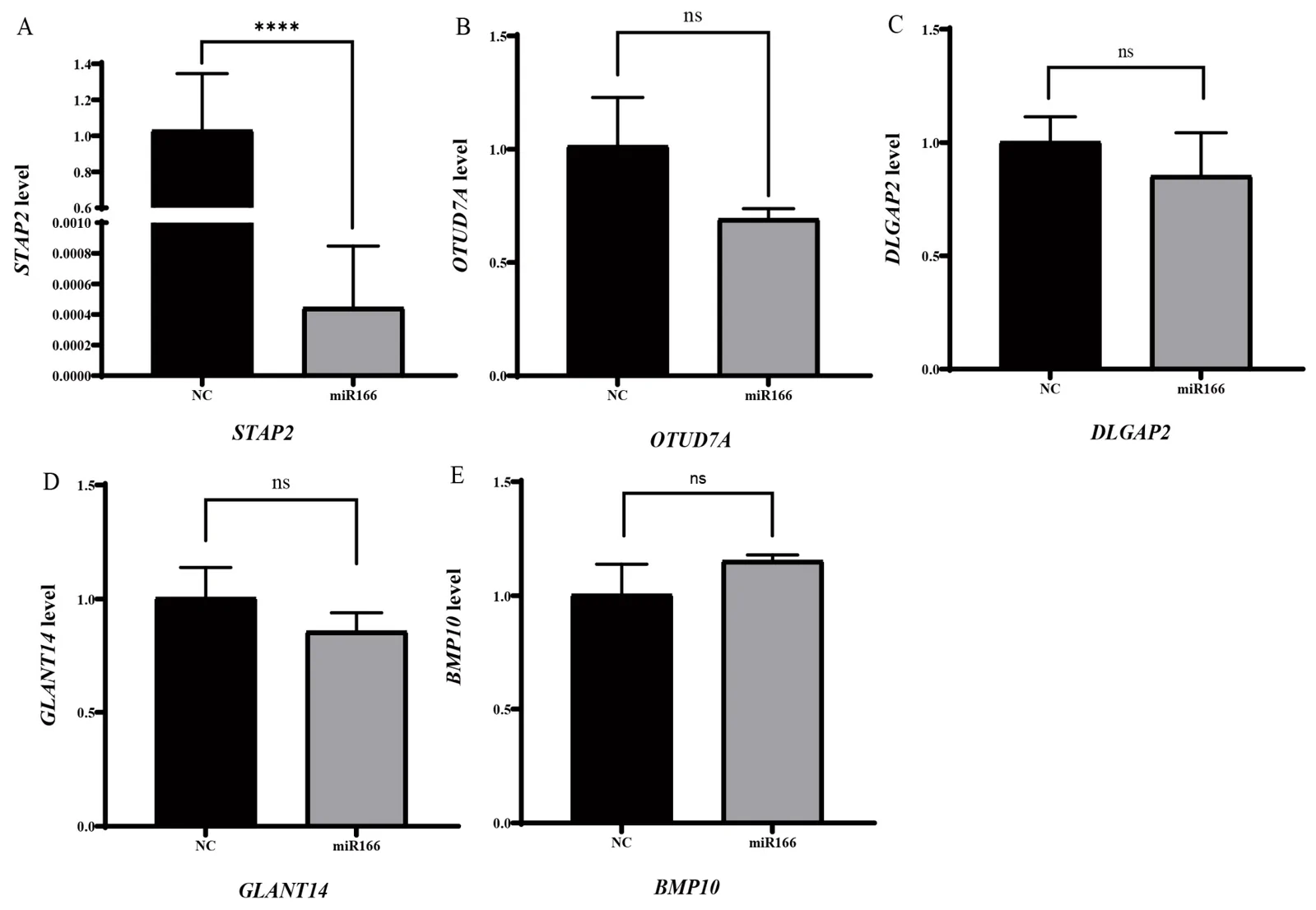
Expression of predicted target genes after transfection of miR166 mimics into HEK293T cells. (**A**) *STAP2*. (**B**) *OTUD7A*. (**C**) *DLGAP2*. (**D**) *GALNT14*. (**E**) *BMP10*. Data are presented as mean ± SD (*n* = 3 biological replicates). Note: ns, not significant; **** *p* < 0.0001 vs. NC group.

**Figure 6:**
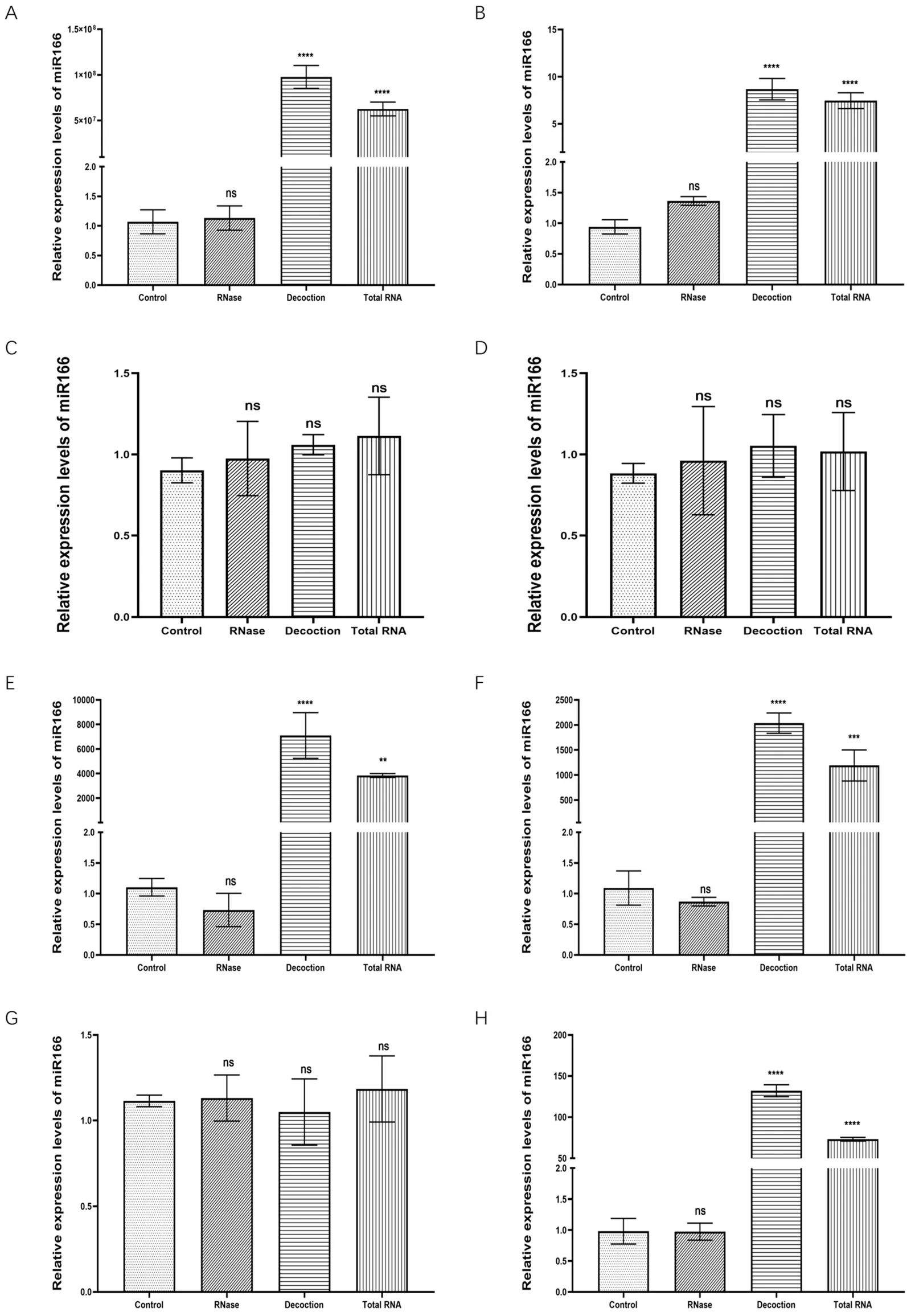
Relative expression of miR166 in different tissues of mice at 1 h post-gavage. (**A**) Stomach. (**B**) Blood. (**C**) Brain. (**D**) Heart. (**E**) Liver. (**F**) Spleen. (**G**) Kidney. (**H**) Intestine. Data are presented as mean ± SD (*n* = 3 biological replicates). Note: ns, not significant; ** *p* < 0.01, *** *p* < 0.001, **** *p* < 0.0001 vs. Control group.

**Figure 7:**
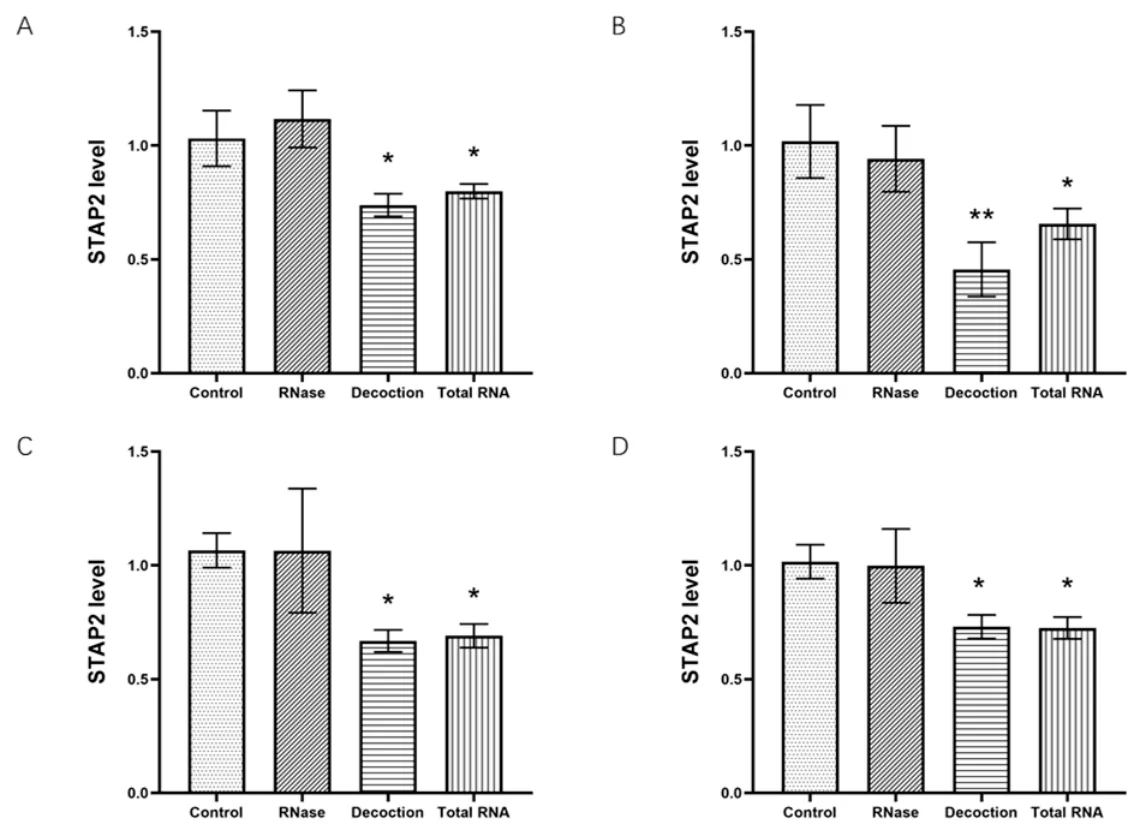
*Stap2* expression in tissues of mice after gavage. (**A**) Blood. (**B**) Liver. (**C**) Spleen. (**D**) Intestine. Data are presented as mean ± SD (*n* = 3 biological replicates). Note: ns, not significant; * *p* < 0.05, ** *p* < 0.01 vs. Control group.

**Figure 8:**
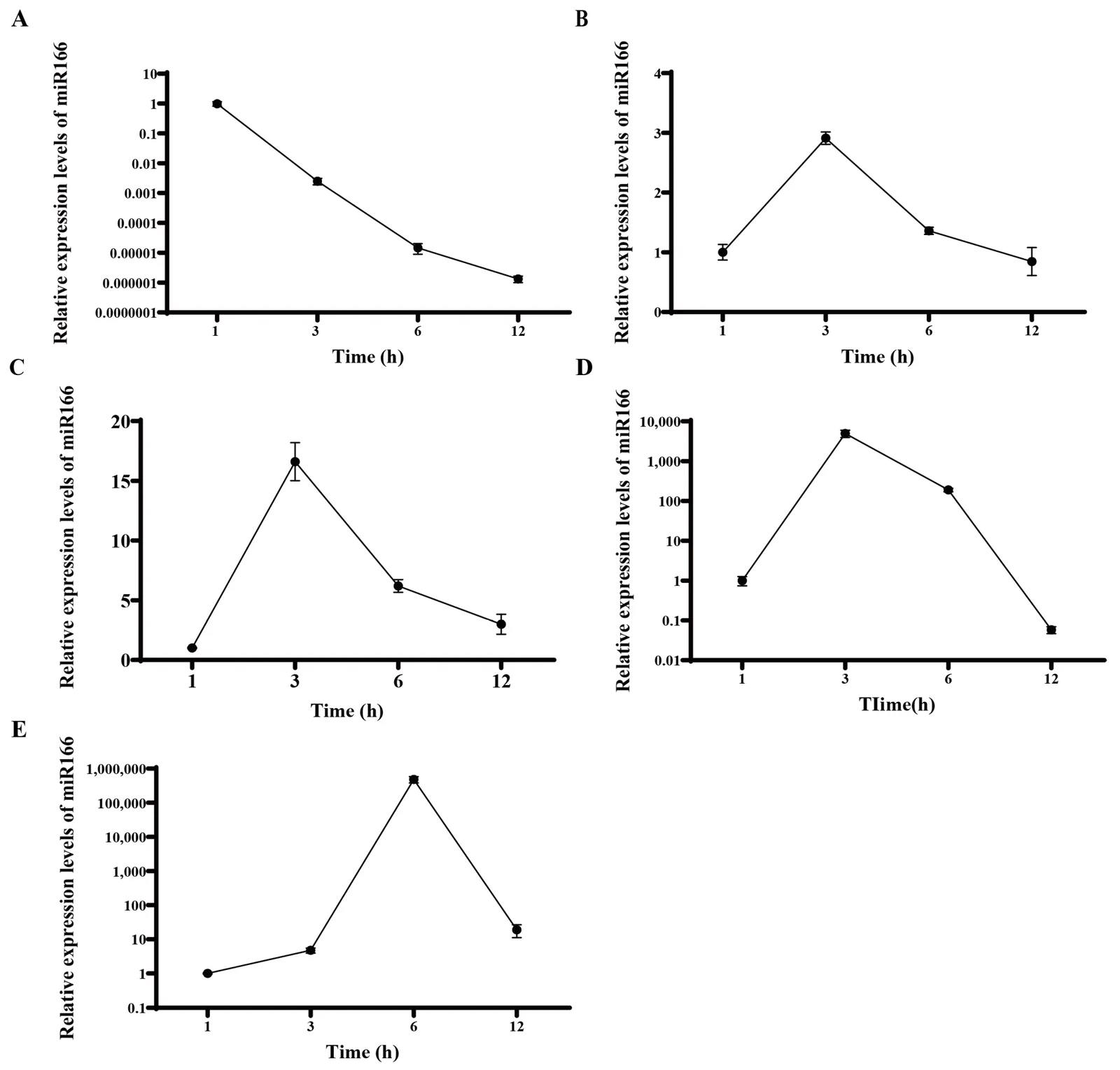
Metabolic kinetics of miR166 in different tissues of mice after gavage of total RNA extracted from fresh *T. mongolicum*. (**A**) Stomach. (**B**) Blood. (**C**) Liver. (**D**) Spleen. (**E**) Intestine. Data are presented as mean ± SD (*n* = 3 biological replicates).

**Figure 9:**
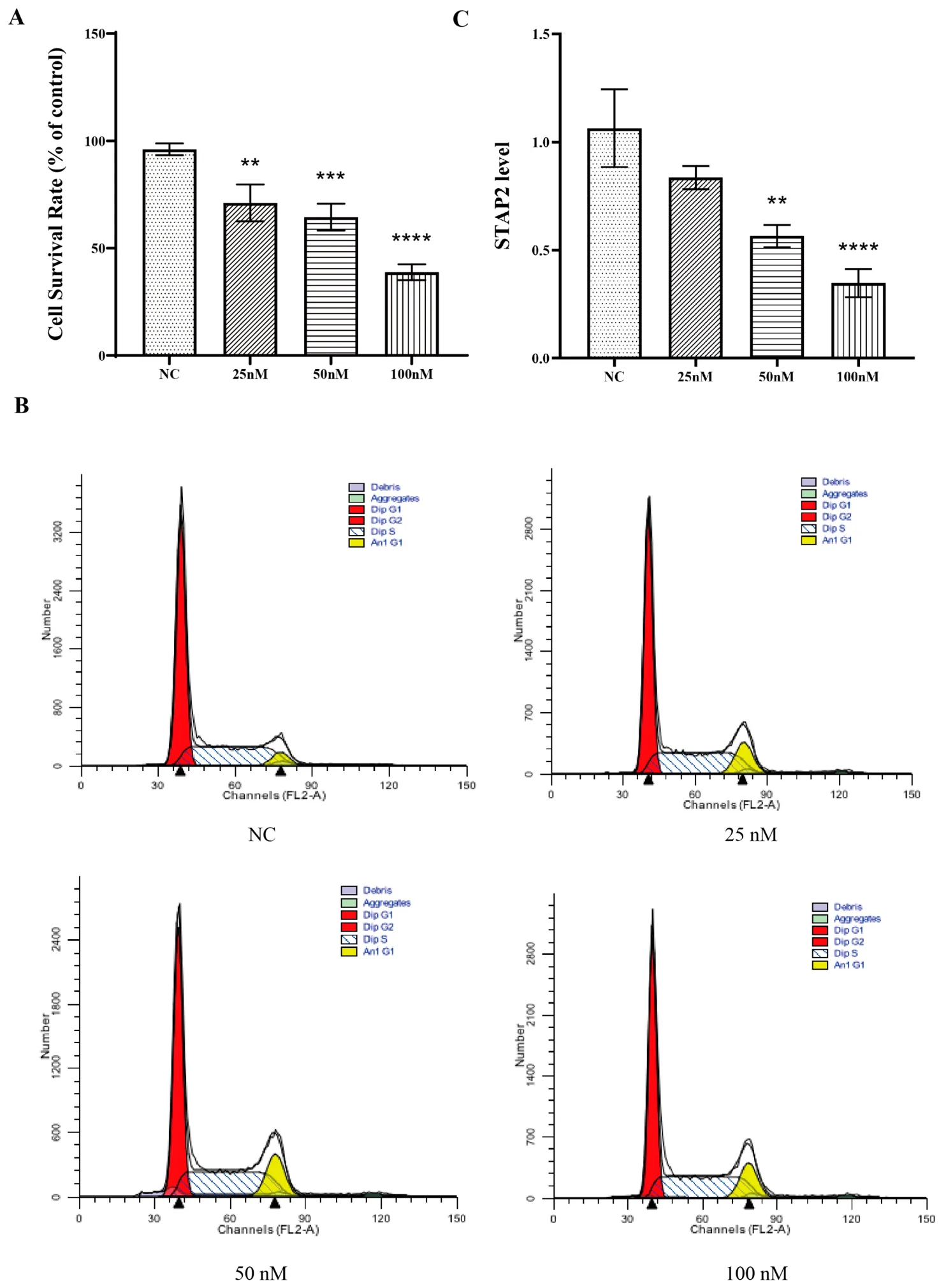
Effects of miR166 on HepG2 cells. (**A**) Cell viability assessed by MTT assay. (**B**) Cell cycle distribution analyzed by flow cytometry. (**C**) Relative *STAP2* expression measured by qRT-PCR. Data are presented as mean ± SD (*n* = 3 biological replicates). Note: ns, not significant; ** *p* < 0.01, *** *p* < 0.001, **** *p* < 0.0001 vs. NC group.

**Table 1:** Primers for Real-time PCR.

Gene Name	Sequences
*OTUD7A* (Human)	Forward: CCTGGAGATGCCAATCTACACA
	Reverse: TGTTGCCTGCTCGATCAAGTC
*DLGAP2* (Human)	CCTCTGACATCACCTCCACCAT
	TCACCGAGATCAGAGGCTTGGA
*GALNT14* (Human)	Forward: CTACGCATCTGATCCGGGAAA
	Reverse: CCAGACCTTGCCGTTCATTATT
*BMP10* (Human)	Forward: ACTGGTGCAAAGGGATCGTAT
	Reverse: AGTCTCCCACTCACTGTTGGT
*STAP2* (Human)	Forward: GACCTTGGAGTGTCGGGAAAT
	Reverse: GAAGCAGGGTCAAGTCGGT
*GAPDH* (Human)	Forward: GGAGCGAGATCCCTCCAAAAT
	Reverse: GGCTGTTGTCATACTTCTCATGG
*Stap2* (Mouse)	Forward: CTACCTCCCCTGCCTCAGTT
	Reverse: CCTGACTGGAGAGAGACACATCC
*Gapdh* (Mouse)	Forward: CCTCGTCCCGTAGACAAAATC
	Reverse: TGAAGGGGTCGTTGATGGC

## Data Availability

The data presented in this study are available in the article and its Supplementary Materials. Raw data are available from the corresponding author upon reasonable request. The raw sequencing data have been deposited in the NCBI Sequence Read Archive (SRA) under BioProject accession number PRJNA1533588.

## Supplementary Materials

The following supporting information can be downloaded at: https://www.mdpi.com/article/10.3390/foods15193470/s1, Supplementary Figure S1. RNase A treatment eliminates RNA in *T. mongolicum* decoction; Supplementary Figure S2. miRNA base preferences; Supplementary Figure S3. Relative miR166 expression levels in HepG2 cells following transfection with different concentrations of miR166 mimics.

## Abbreviations

The following abbreviations are used in this manuscript:

**Abbreviation**	**Full form**
FT	Fresh *T. mongolicum*
DT	Decoction of *T. mongolicum*
GO	Gene Ontology analysis
KEGG	Kyoto Encyclopedia of Genes and Genomes
CPM	counts per million
PBS	phosphate-buffered saline
MTT	3-(4,5-dimethylthiazol-2-yl)-2,5-diphenyltetrazolium bromide
HBsAg	hepatitis B surface antigen
NC	negative control
BMP10	bone morphogenetic protein 10
DLGAP2	DLG associated protein 2
GALNT14	polypeptide N-acetylgalactosaminyltransferase 14
OTUD7A	OTU deubiquitinase 7A
PI3K	phosphatidylinositol 3-kinase
GAPDH	glyceraldehyde-3-phosphate dehydrogenase
STAP2	signal transducing adaptor family member 2
STAT3	signal transducer and activator of transcription 3
DMEM	Dulbecco’s Modified Eagle Medium
DMSO	dimethyl sulfoxide
FBS	fetal bovine serum
DUSP12	dual specificity phosphatase 12
