# Supplementary material for: A Novel Cross-Kingdom Regulatory Mode of Thermostable miR166 from Taraxacum mongolicum Inhibits HepG2 Cell Proliferation in Association with Downregulation of the Candidate Target STAP2
Source: Foods. 2026 Sep 28;15(19):3470. doi: 10.3390/foods15193470 (PMC13664370; doi:10.3390/foods15193470)
Supplement: Supplementary file 1 [file foods-15-03470-s001.zip › foods-4585325-supplementary.pdf]

# Supplementary Material

**Supplementary Figure S1. RNase A treatment eliminates RNA in *T. mongolicum* decoction.**

**Supplementary Figure S2. miRNA base preferences.**

**Supplementary Figure S3 Relative miR166 expression levels in HepG2 cells following transfection with different concentrations of miR166 mimics.**

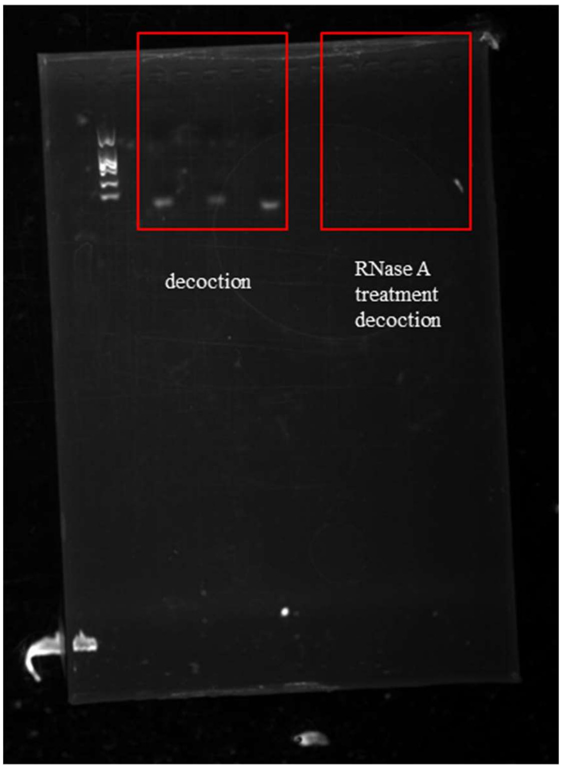


**Supplementary Figure S1. RNase A treatment eliminates RNA in T. mongolicum decoction.**

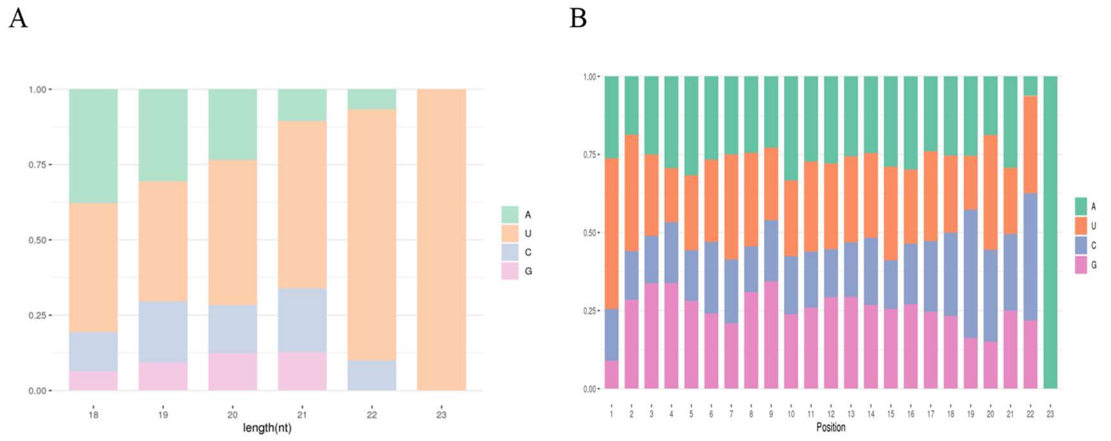


**Supplementary Figure S2. miRNA base preferences.** (A) Preferential distribution of first bases in miRNAs of different lengths. (B) Statistic analysis of base preferences at each site in miRNAs.

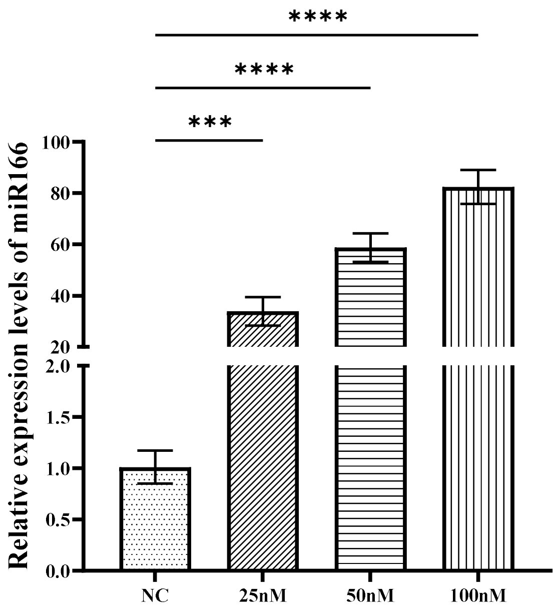


**Supplementary Figure S3. Relative miR166 expression levels in HepG2 cells following transfection with different concentrations of miR166 mimics.** HepG2 cells were transfected with miR166 mimics at concentrations of 25, 50, and 100 nM, or negative control (NC) mimics. Data are presented as fold change relative to the NC group (mean ± SD, n = 3).

Note: * P < 0.05; ** P < 0.01; *** P < 0.001, **** P<0.0001.
